# The effect of adding Rosmarinic and Ascorbic acids to vitrification media on fertilization rate of the mice oocyte: An experimental study

**DOI:** 10.18502/ijrm.v17i3.4518

**Published:** 2019-05-29

**Authors:** Abdollah Borjizadeh, Hamid Ahmadi, Erfan Daneshi, Daem Roshani, Fardin Fathi, Mahdad Abdi, Sherko Nasseri, Morteza Abouzaripour

**Affiliations:** ^1^Department of Anatomy, Faculty of Medicine, Kurdistan University of Medical Sciences, Sanandaj, Iran.; ^2^Department of Anatomy, Faculty of Medicine, Tehran University of Medical Sciences, Tehran, Iran.; ^3^Social Determinants of Health Kurdistan Research Center, Kurdistan University of Medical Sciences, Sanandaj, Iran.; ^4^Cellular and Molecular Research Center, Kurdistan University of Medical Sciences, Sanandaj, Iran.

**Keywords:** *Vitrification*, * Antioxidant*, * Fertilization.*

## Abstract

**Background:**

Oocytes vitrification is a pivotal step for the widespread and safekeeping of animal genetic resources. Oocytes endure notable morphological and functional damage during cryopreservation. Oxidative stress is one of the adverse effects that vitrification imparts on oocytes.

**Objective:**

In the present study, we investigated the antioxidant effect of Rosmarinic and Ascorbic acids on the quality and fertilizing ability of frozen-thawed mice oocyte.

**Materials and Methods:**

In this experimental study, germinal vesicle oocytes obtained from two-months-old (30–40gr) NMRI mice were randomly divided into four groups. The basic cryoprotectants were 7.5% (v/v) ethylene glycol+7.5% (v/v) Propanediol as an equilibration media. Vitrification medium contained 15% (v/v) ethylene glycol+15% (v/v) propanediol, and 0.5 M sucrose. In the first group (Control), nothing was added to vitrification mediums, whereas, in the second and third groups, 0.5 mmol/L of Ascorbic acid and 105 µmol/L of Rosmarinic acid were added into vitrification medium, respectively. The cumulative concentration of Rosmarinic and Ascorbic acids were added to group 4. Mouse oocytes were vitrified and preserved for one month. The thawed oocytes were transferred into the α-MEM medium (Alpha Minimum Essential Medium) and maintained in this medium for 24 hr, to be matured and reach the metaphase II stage.

**Results:**

The addition of Rosmarinic and Ascorbic acids to the vitrification solution improved the survival, maturation of Germinal vesicles, fertilization rate, and finally development to 4-cell stage. Maturation rates to 4-cell stage for Ascorbic acid, Rosmarinic acid, and both of them together were 80%, 80.76%, and 86.61%, respectively.

**Conclusion:**

These results indicate that the addition of a cumulative concentration of 0.5 mmol/L Ascorbic acid and 105 µmol/L of Rosmarinic acid to the cryopreservation solution for the mouse immature oocytes would be of significant value (p< 0.01).

## 1. Introduction

Successfully oocytes cryopreservation with minority loss in viability is crucial for the success of assisted reproductive technologies (1). Despite recent advances in vitrification methods which results in high pregnancy rates and live births, Cryoprotectants toxicity is the hot topic of cryobiology studies (2). Oxidative stress is one of the adverse effects that cryopreservation imparts on oocytes, it has been assumed that oocytes without a potent defense system to preserve their delicate cellular structure are exposed to free radicals in cryoprotectants (3). Reactive oxygen species (ROS) induce lipid peroxidation which adversely affects membrane structure, fluidity, and function (4). It has also been proposed that the repair of cell structures requires energy generation with a subsequent rise in ROS production (5). Evidently, the post-thaw survival and fertilization rate of oocytes after cryopreservation remains low (6). Therefore, the main challenges are to find optimal Cryoprotectants solutions. It is feasible that the inclusion of an antioxidant in the cryoprotectants helps in maintaining oocyte viability after the cryopreservation procedure by reducing the effects of harmful oxygen radicals (7). A variety of antioxidants have been studied before, but duad supplementation with Rosmarinic acid (RosA) and Ascorbic acid (AA) is our experience. In classical medicine, *Rosmarinus officinalis* with its Phenolic compounds are widely used for the treatment of diabetes, pulmonary diseases, nerve complication, and inflammatory diseases. Today it has been shown that Rosmarinous officinalis contains antioxidant molecules, including RosA, carnasol, and diphenol rosemary (8, 9). Phenolic compounds have various biological functions such as oxidation inhibitory function, preventing DNA from mutation and oxidation and anti-thrombotic effects (10). Apart from RosA, AA which accumulates highly in ovaries (theca, granulosa, and luteal cells) and other endocrine tissues has a significant antioxidant function (11). This antioxidant, which significantly decreases granulosa cells and preantral follicles apoptosis in vitro (12), play an important role in fertility preservation and also increases the integrity and stability of cell membrane (13). AA has many biological actions of particular relevance to reproduction, each dependent on its role as a reducing agent. It is essential to prevent or diminish the oxidation of biomolecules due to its capability to donate electrons; it is essential for the biosynthesis of collagen, crucial for follicular growth, ovulation, and corpus luteum formation. Additionally, AA positively influences the process of steroidogenesis (14, 15).

To our knowledge, the antioxidant effects of RosA and AA from natural sources on NMRI germinal vesicle cryopreservation has not been evaluated to date. So we decided to investigate the protective effects of simultaneous supplementation of RosA and AA into a vitrification medium of GV oocytes on the survival, viability, maturation, fertilization, and development to cleavage stage embryos followed by thawing.

## 2. Materials and Methods

### Oocytes preparation

Supraovolution were induced by an intraperitoneal (IP) injection of 10 IU of pregnant mare's serum gonadotropin PMSG. In order to retrieve GV oocytes, 48 hr after the PMSG injection, the mice were sacrificed by cervical dislocation. Ovaries were removed under sterile conditions and transferred into Ham's culture medium containing N-2-Hydroxyethylpiperazine-N'-2-Ethanesulfonic Acid HEPES buffer, 10% Fetal Bovine Serum, and 1% of the combination of penicillin and streptomycin antibiotics. After washing and removing connective tissue and surrounding fat, the ovaries were examined under a stereomicroscope, and the GV-containing oocytes with granulosa cells were collected from the ovaries (6).

### Experimental groups

The collected GV-stage oocytes obtained from 2-months-old NMRI mice were randomly divided into control and experimental groups. Group one was considered as control and nothing was added to the vitrification medium. In second, third, and forth groups, AA (0.5 mmol/L) (7), RosA (105 µmol/L) (13), and Accumulative AA and RosA were added into vitrification medium, respectively. In this experimental study, the animals were kept under controlled conditions (12 hr light/dark), fed with water ad libitum. All reagents were purchased from Sigma-Aldrich Co., USA.

### Reagents preparation 

The basic cryoprotectants were 7.5% (v/v) ethylene glycol+7.5% (v/v) Propanediol as an equilibration media. Vitrification medium was containing 15% (v/v) ethylene glycol+15% (v/v) Propanediol, and 0.5 M sucrose. In the first group (Control), nothing was added to vitrification mediums, whereas, in the second and third groups, 0.5 mmol/L of AA and 105 µmol/L of RosA were added into vitrification medium, respectively. The cumulative concentration of Rosmarinic and Ascorbic acids were added to group 4 (7, 13).

### Vitrification and thawing protocols

Briefly, the germinal vesicles with cumulus cells were placed in equilibration medium containing 7.5% (v/v) ethylene glycol (EG)+7.5% (v/v) Propanediol (PROH) for 5 min at room temperature, and were then transferred to vitrification medium containing 15% (v/v) EG+15% (v/v) PROH, and 0.5 M sucrose at room temperature for 45 to 60 sec. They were loaded on cryotube and were plunged immediately into LN2 for at least 1 month of storage. For the thawing, after picking up germinal vesicles from nitrogen tank, the cryotube containing the oocytes were first stored for 10 sec on a liquid nitrogen vapor and then placed in 37${}^{\circ }$C warm water for 10 sec. At the second step, the contents of the cryotube were transferred into the thawing droplet containing 1 M sucrose and 20% HAS for 1-2 min. Then, they were transferred into a diluents solution containing 0.5 and 0.25 molar of sucrose with 20% human serum albumin and were kept for 3 min in each one, respectively. Subsequently, in order to efflux the cryoprotectants from the oocytes, they were introduced into the PBS droplets for 3 min. After these steps, the thawed oocytes were transferred into the α-MEM medium (Alpha Minimum Essential Medium) and maintained in this medium for 24 hr, to be matured and reached the metaphase II stage. The viability of GV and the number of MII stage embryos were assessed in each group (16).

### Sperm preparation and in vitro fertilization (IVF)

NMRI male mice at 8 weeks of their age were sacrificed by cervical dislocation. The cauda epididymis was removed and transferred into 500 μl of HTF medium containing 10 mg/ml (BSA10). The specimens were then placed in an incubator at 37${}^{\circ }$C with 5% CO${}_{2}$ for 1–1.5 hr to be capacitated. Then the concentration, morphology, and motility of the sperm were assessed using a microscope. In order to do IVF, about 1×10${}^{6}$/ml sperm were added into each HTF droplets containing about 100 MII oocytes. Then they were incubated at 37${}^{\circ }$C under 5% CO${}_{2}$, 5% O${}_{2}$ balanced in 90% N${}_{2}$. After 8 hr of co-incubation with spermatozoa, the oocytes were examined for the presence of male and female pronuclei under an inverted microscope. Presence of pronuclei guided us to 2pn cells.

The fertilized zygotes transferred into preincubated KSOM medium and were stored to reach the 4-cell stage and then were transferred into fresh KSOM medium droplets. The number of embryos at 2- and 4-cell stage were counted in different groups and compared (6).

### Ethical consideration

All procedures were performed in accordance with the approval of the Institutional Animal Care and Use Committee at the Kurdistan University of Medical Sciences, Sanadaj, Iran (code: 22725).

### Statistical analysis

All statistical analyses were performed using SPSS version 16. The effect of Ascorbic acid, Rosmarinic acid, and Ascorbic acid together with Rosmarinic acid on survival and viability of GV, maturation to MII oocyte, fertilization rate, development to 2-cell and 4-cell stages were analyzed by the analysis of variance (ANOVA). Data are presented as a mean ± standard deviation. A p < 0.01 was considered as statistically significant.

## 3. Results

As shown in Table I, in the control group, the viability and survival rate (76.76%) was lowest compared to other experimental groups. The viability and survival rate was highest (90.94) in group 4 (Ascorbic with Rosmarinic acids) and the difference was statistically significant in comparison with other experimental groups. Also, there were statistical differences in viability and survival among AA and RosA groups separately compared to control groups (p > 0.01).

The results showed that the maturation rate from GV into MII oocyte (82.35%) and development into 2-cell stage (69.78%) were highest in AA with RosA in comparison to the remaining three groups, and the differences are statistically significant (p > 0.01). These criteria are higher in groups 2 and 3 in comparison to the control group, but the differences are not statistically significant.

**Table 1:** Viability and the number of GV, MII oocytes, 2-cell, and 4-cell stages embryos and fertilization rate in different experimental groups


**Groups**	**Vitrified GV**	**Viability of GV**	**MII oocytes**	**2-cell stage**	**4-cell stage**
Control	198	152 (76.76%)	105 (69.07%)	54 (51.42%)	41 (75.92%)
AA	239	203 (84.93%)	153 (75.36%)	90 (58.82%)	72 (80%)${}^{\#}$
RosA	228	193 (84.69%)	138 (71.50%)	78 (56.52%)	63 (80.76%)${}^{\wedge }$
AA + RosA	243	221 (90.94%)	182 (82.35%)	127 (69.78%)	110 (86.61%)${}^{\$}$
Data presented as Mean ± SD; analysis of variance (ANOVA)					
Note: #: p < 0.01 vs. Control, RosA and AA+RosA; ∧: p < 0.01 vs. Control, RosA and AA+RosA; $: p < 0.01 vs. Control, RosA and RosA; RosA: Rosmarinic acid; AA: Acid ascorbic; GV: Germinal vesicle; M II: Metaphase2; there are no significant differences in development to 4-cell stage among experimental groups.					

## 4. Discussion

Our findings suggested that AA and RosA could improve the viability and survival of GV, maturation of GV to MII oocyte, and also development into 2-cell stage, but when this oxidant combined together, they could exert strong potential effects on the mentioned characters of oocytes and embryos.

It seems that ROS are an inseparable part of gamete function. During IVM, the formation of ROS with the hypoxanthine-xanthine oxidase system, in the presence of AA and RosA, can enhance the developmental potential of oocytes to produce embryo (17).

The process of vitrification and thawing increases the production of ROS; manifold researches have focused on the effect of antioxidants as regards to boosting the quality of post-thaw oocytes. Indeed there is some evidence that the addition of antioxidants to cryoprotectants diminishes the adverse effects of ROS. Oxygen radicals and ROS play both physiological and pathological roles in the female reproductive tract. Normally the pathologic effects are exerted by various mechanisms, including lipid damage, inhibition of protein synthesis, and depletion of ATP (18). The oxidative stress may enforce the distribution of cytochrome c and other apoptogenic factors from cell mitochondria which finally activates apoptosis (19, 20). Therefore, in order to reduce such damage, improvement of the solutions used in cryopreservation is needed. There are potent proofs that injuries caused by lipid peroxidation can be diminished by the use of antioxidants. The main objective of the present study was to evaluate the effect of using antioxidant including AA, RosA, and duad supplementation of them to the vitrification medium of GV on viability, survival, maturation, fertilization, and development of oocytes and embryos following freeze and thaw process. In this study, 105 µmol/L of RosA and 0.05 mmol/L of AA was added into the vitrification medium as optimal concentrations. It has been shown that adding 0.1 mmol/L of AA into freezing medium could improve the development of the embryo in vitro (7), we reached into same results with different dose (0.5mmol/L), maybe because of using different cryoprotectants and GV vitrification instead of an embryo. The presence of AA in follicular fluid (19) indicates that it may have a physiological role as an antioxidant in oocyte and embryo development. Ascorbate is a very potent antioxidant that is able to scavenge ROS products in vivo and in vitro. AA ceases per oxidative process due to its capability to give electrons from both the second and third carbon (21). AA also helps recycle oxidized vitamin E and glutathione (22), and it is a cofactor in the biosynthesis of L-carnitine – a molecule required for the oxidation of fatty acids (23). L-carnitine has been used functionally to alleviate embryo lipid content and modify cryotolerance of embryos (24). Another strong reducing agent is the RosA. Researchers suggested that RosA at a concentration of 70–100 µmol/L can exert a strong antioxidant effect on sperm (13). That is compatible with our findings, which showed that adding 105 µmol/L of RosA results in the improvement of GV maturation and MII fertilization rate. In addition, it has been demonstrated that adding a different concentration of RosA to sperm freezing medium improved the motility, cell membrane and acrosomal integrity, peroxidation rate, and fertilization potential following thawing (13). The mechanism underlying the function of rosemary on cryopreservation of oocyte is still unclear, but it is demonstrated that cryoprotectants supplementation with rosemary resulted in a reduction of ROS reaction and decreasing malonaldehyde (MDA) levels after cryopreservation (13). In RosA, carboxylic acid group together with the catechol elements in the aromatic ring are responsible for neutralizing free radicals. Since antioxidants entrap the free radicals and protect the cells against oxidative stress so that adding them into culture medium can exert positive effects on cell survival and development (16).

In the present study for the first time, RosA was added to oocyte vitrification medium and improved the survival, viability rate of GV, and also the fertilization rate of MII oocytes.

It seems that AA reduced per oxidative process due to its capability to give electrons and RosA has neutralized free radicals.

## 5. Conclusion

The addition of AA (0.5 mM) plus RosA (105 µmol/L) in the vitrification medium improves the survival of in vitro-produced embryos.

##  Conflict of Interest

The authors declare that there is no conflict of interest.
